# Testicular Dirofilariasis in an Italian 11-Year-old Child

**DOI:** 10.1097/INF.0000000000003706

**Published:** 2022-09-08

**Authors:** Andrea Pansini, Vittoria Carlotta Magenes, Francesca Casini, Gabriella Guida, Marina De Sanctis, Carlotta Paola Maria Canonica, Roberta Simona Rossi, Gianvincenzo Zuccotti, Vania Giacomet

**Affiliations:** From the * Paediatric Surgery Department, Vittore Buzzi Children’s Hospital; † Paediatric Department, Vittore Buzzi Children’s Hospital, Università di Milano; ‡ Paediatric Infectious Disease Unit, Università di Milano, ASST FBF-Sacco, Milan, Italy; § Primary Care Paediatrician ASST Ovest Milan, ATS MILAN; ¶ Pathology Unit, Università di Milano, ASST FBF-Sacco, Milan, Italy.

**Keywords:** Italy, testicular dirofilaria

## Abstract

Dirofilariasis is a rare infection caused by a vector-borne nematode that can be accidentally transmitted to humans. We report a case of a 11-year-old child with a painless scrotal cyst caused by *Dirofilaria repens*, initially suspected by ultrasound scan and then confirmed by histopathologic examination.

## CASE REPORT

A Caucasian 11-year-old boy was referred to our institution in December 2021 with a 4 months history of nodular mass in the right scrotum. The nodule, discovered by chance, was painless and was not associated with any other symptom or testicular discomfort. The clinical examination revealed a mass in the right scrotum measuring about 1 cm, the skin above and around the nodule did not show signs of inflammation. A urologic examination was negative for hydrocele, varicocele, testicular or epididymal abnormalities, peripheral lymphedema and adenopathy. His past medical history was unremarkable. No fever or signs of systemic infection were detected. During the preceding weeks, the child had no signs or symptoms of infectious diseases nor he had assumed any drugs. Moreover, no previous travels to regions endemic for filariasis were reported, and no contact with animals or tick bites were referred; no parasites were found in the stool.

Ultrasonography (US) detected a 7 mm cystic inhomogeneous hypoechoic mass in the right scrotum. At US examination, the mass was near but not apparently adherent to the testis or the epididymis. The cyst was between the tunica albuginea and the subcutaneous tissue, and it had a rich peripheral vascularity.

**FIGURE 1.**
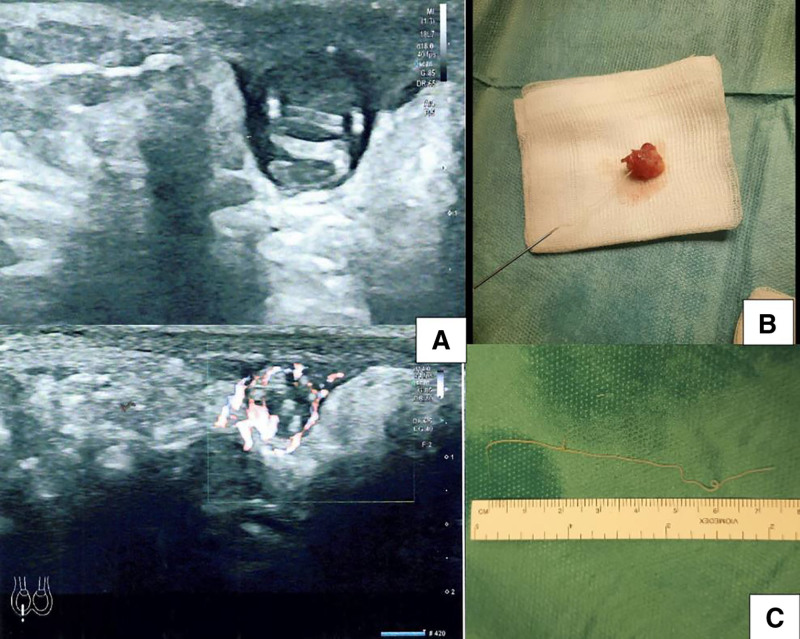
Ultrasonographic and macroscopic aspect of *Dirofilaria repens*. A: Scrotal ultrasound showing a 8 × 10 mm cyst with peripheral vascularity containing echogenic linear undulating structures. These structures have the peculiar movements characteristic of the filarial dance sign on real-time ultrasonography; (B) the nematode was extracted intact alive from the cystic nodule. It shows spontaneous movements; (C) mature female worm of Dirofilaria repens.

The patient’s condition was closely monitored, and the scrotal US was repeated after 2 months. US imaging of the scrotal cyst showed a fusiform, elongated and mobile hyperechoic structure suspected for “filarial dance sign” (Fig. 1A).

A surgical approach was considered and excision of the lesion for microscopical and histologic assessment was recommended.

Surgical excision was performed under general anesthesia by a scrotal incision over the nodule. The mass was not in connection with testicular or funicular structure and was separated with sharp dissection to the surrounding tissue. It was enucleated intact (Fig. 1B). A long thin worm popped out after puncture of the cyst.

The morphologic examination of the worm showed that it was a mature female of 80 mm in length and 1 mm in width (Fig. 1C).

The anterior and posterior ends of the body were slightly narrowed and had a roundish shape. Pectiniform longitudinal cross striations were observed.

The macroscopic histologic examination revealed a filamentous structure of 6 cm of length and 0.1 cm of diameter, instead the microscopic evaluation showed a parasitic pseudocyst associated with a filarial nematode identified as *Dirofilaria repens*, without evidence of granuloma or eosinophilic infiltrates. Cystic fluid examination was negative for microfilariae.

An abdominal US was performed and resulted normal.

Routine laboratory tests of blood were normal (white blood count 7400/mmc, hemoglobin 13.8 g/dL, platelts 254,000, polymerase chain reaction negative), no evidence of eosinophilia (190/L, normal value 30–510, 2.6%), IgE 7.24 kU/L (normal value < 100).

First-level immunologic examinations were normal for age, only IgM were at the lower limit of normal (10.30 g/L, normal value 0.40–2.3 g/L), while serologic examination for *Echinococcus* spp., *Toxocara* spp. as Quantiferon blood assay tests for tuberculosis and parasitologic fecal examinations were negative.

In addition, an ophthalmologic evaluation excluded parasitic localization within the eye.

## DISCUSSION

We report a new case of testicular dirofilariasis in a pediatric patient, complaining of a painless scrotal nodule without testicular pain nor any other signs or symptoms. Human dirofilariasis is a zoonotic infection caused by nematode worm *D. repens* or, less frequently, *D. immitis*. Definitive hosts and reservoirs of the parasites are domesticated and wild dogs, foxes, racoons, rarely cats, and the most common vectors are insects of the family *Culicidae*, genera *Anopheles*, *Aedes* and *Culex*.^1^ Dogs are the main source of infection and humans are accidental hosts.^1^ Humans are dead-end hosts in whom the nematode cannot reproduce.^1^ *Dirofilaria repens* is endemic around the Mediterranean area and it has been recently considered as an emerging metazoonosis in southern Europe. Italy is the country with the greatest number of reported human cases.^2,3^ Interestingly, Gabrielli et al^4^ recently described 8 cases, observed in Central Italy during the years 2018–2019. In this article, no pediatric patients were described, nor scrotal localization was found.^4^ Defining the exact prevalence of human dirofilariasis is challenging, as the disease tends to be asymptomatic and the physicians may not be aware of the condition, leading to an underestimated number of cases.^4^

The usual clinical presentation of *D. repens* in humans is either a subcutaneous nodule (70%) or a conjunctivitis in 30%.^5^ Although few cases of scrotal dirofilariasis have been previously reported in adults, this condition remains a rare event described in pediatric age.^6,7^ Two cases were reported in Italy. A 3-year-old child underwent orchiectomy as infestation by *Dirofilaria repens* was mimicking an acute scrotum.^8^ The other one, a 11-month-old child evaluated for suspicion of neoplasm presenting with an asymptomatic scrotal mass.^9^ In dirofilariasis, no reliable signs, symptoms or laboratory parameters are available. Eosinophilia and elevated IgE levels are almost always absent as in our case, although elevated IgE levels could confirm the diagnostic suspicion.^10^ In our case, US examination was suggestive of parasitosis. Surgical excision, which is the treatment of choice, was performed, and histopathologic analysis confirmed the final diagnosis. Given the complete surgical excision and the exclusion of other localizations, no pharmacologic therapy was necessary.

Dirofilariasis should be regarded in the differential diagnosis of asymptomatic testicular mass. It actually can mimic solid scrotal masses, from which it can be distinguished by clinical history and ultrasound scan.^9^ Moreover, dirofilariasis must be differentiated from other helminth infections since IgE levels and eosinophilia are usually absent.^10^

This case illustrates the importance of searching for a helminthic etiology in cystic mass displaying US characteristics of motile tubular structures at testicular level or at any other location. Importantly, a clear and thorough medical, social and familial recent history should be taken, bearing in mind that neither a negative history for travels to regions endemic for parasitosis or the absence of animals or tick bites exclude the infection. Epidemiologic studies are needed to provide further evidence concerning the incidence of human dirofilariasis in Italy.
